# Suppression of ERK signalling abolishes primitive endoderm formation but does not promote pluripotency in rabbit embryo

**DOI:** 10.1242/dev.156406

**Published:** 2017-10-15

**Authors:** Anna Piliszek, Zofia E. Madeja, Berenika Plusa

**Affiliations:** 1 Department of Experimental Embryology, Institute of Genetics and Animal Breeding, Polish Academy of Sciences, Postępu 36a, 05-552 Jastrzębiec, Poland; 2 Department of Genetics and Animal Breeding, Faculty of Veterinary Medicine and Animal Sciences, Poznan University of Life Sciences, Wołyńska 33, 60-637 Poznań, Poland; 3 Faculty of Biology, Medicine and Health, The University of Manchester, Oxford Road, Manchester M13 9PT, UK

**Keywords:** Blastocyst, Epiblast, FGF, Primitive endoderm, Rabbit

## Abstract

Formation of epiblast (EPI) – the founder line of all embryonic lineages – and extra-embryonic supportive tissues is one of the key events in mammalian development. The prevailing model of early mammalian development is based almost exclusively on the mouse. Here, we provide a comprehensive, stage-by-stage analysis of EPI and extra-embryonic primitive endoderm (PrE) formation during preimplantation development of the rabbit. Although we observed that rabbit embryos have several features in common with mouse embryos, including a stage-related initiation of lineage specification, our results demonstrate the existence of some key differences in lineage specification among mammals. Contrary to the current view, our data suggest that reciprocal repression of GATA6 and NANOG is not fundamental for the initial stages of PrE versus EPI specification in mammals. Furthermore, our results provide insight into the observed discrepancies relating to the role of FGF/ERK signalling in PrE versus EPI specification between mouse and other mammals.

## Abstract (precis)

**Highlighted Article:** A comprehensive analysis of rabbit preimplantation development reveals key differences between rabbit and mouse, with some aspects of lineage specification in rabbit more closely resembling that of human and primate embryos.

## INTRODUCTION

Preimplantation development in the mammalian embryo is characterised by two consecutive cell fate specification events. During the first, cells located on the outside of the embryo polarise and form an extra-embryonic epithelial layer, the trophectoderm (TE), encapsulating the whole embryo. The apolar inner cells are displaced to one side of the embryo by the expansion of the fluid-filled cavity and form the inner cell mass (ICM) (Smith and McLaren, 1977). TE gives rise to the embryonic part of the placenta, whereas ICM cells further differentiate into two lineages: the pluripotent epiblast (EPI) and a second extra-embryonic lineage, the primitive endoderm (PrE) (also called hypoblast). EPI cells are the precursor lineage for the embryo proper, whereas PrE cells give rise to endoderm of the yolk sac and also contribute to definitive endoderm later in development (reviewed by Chazaud and Yamanaka, 2016). Failure in specification of any of these lineages results in developmental arrest and/or early pregnancy loss.

In the murine embryo, at the blastocyst stage, EPI is characterised by the expression of pluripotency markers such as NANOG, SOX2 and OCT4 (also known as POU5F1) (Avilion et al., 2003; Chambers et al., 2003; Kirchhof et al., 2000; Mitsui et al., 2003; Nichols et al., 1998; Scholer et al., 1990), whereas PrE-specific transcription factors, in order of sequential activation, include GATA6, SOX17, GATA4 and SOX7 (Artus et al., 2011; Koutsourakis et al., 1999; Niakan et al., 2010; Schrode et al., 2014; Soudais et al., 1995). However, at the onset of the first lineage differentiation event, at the morula stage, all cells co-express the respective PrE and EPI markers GATA6 and NANOG. After blastocyst formation, progressive restriction of gene expression to the lineage precursors is initiated. In a subset of ICM cells, NANOG expression becomes gradually downregulated whereas GATA6 expression is maintained, leading to specification of the PrE lineage. The remaining cells retain NANOG expression, downregulate GATA6, and specify as the EPI lineage (Chazaud et al., 2006; Plusa et al., 2008; Bessonnard et al., 2014). These events result in establishment of a mutually exclusive EPI- or PrE-specific transcriptional profile among ICM cells. At this stage, EPI and PrE progenitors are positioned within the ICM in an apparently random, salt-and-pepper manner (Chazaud et al., 2006). Concomitantly, EPI- and PrE-specific factors are downregulated in TE cells whereas expression of TE-specific factors like CDX2 and GATA3 is upregulated in these cells (reviewed by Saiz and Plusa, 2013). The subsequent sorting of the EPI and PrE precursors results in the formation of a uniform layer of PrE epithelium that separates the EPI from the blastocyst cavity (Gardner and Rossant, 1979).

The current model of EPI-versus-PrE specification, based on mouse studies, proposes that activation of the FGF/extracellular signal-regulated protein kinase (ERK) pathway directs some ICM cells towards a PrE fate, whereas cells irresponsive to FGF/ERK pathway stimulation contribute to the EPI (Feldman et al., 1995; Goldin and Papaioannou, 2003; Grabarek et al., 2012; Kang et al., 2013; Krawchuk et al., 2013; Nichols et al., 2009; Yamanaka et al., 2010).

Although all eutherians progress through similar developmental stages during preimplantation development, a growing body of evidence suggests considerable differences in early lineage specification between species (reviewed by Frankenberg et al., 2016), with human, bovine, pig and rabbit embryos sharing several properties that are absent in mouse embryos (reviewed by Kuijk et al., 2015; Piliszek et al., 2016). Recent meta-analysis of mRNA transcripts in mouse and human embryos revealed the existence of significant interspecies differences in gene expression dynamics during the preimplantation period (Blakeley et al., 2015). In contrast to data obtained in mouse, treating human embryos with FGF/ERK inhibitors failed to prevent PrE formation (Roode et al., 2012). This questions the evolutionary robustness of mechanisms of early lineage specification in mammals.

To gain insight into how pluripotency is established in non-rodent mammals, we sought to analyse the specification of ICM lineages in the rabbit. Detailed analysis of the localisation of the key pluripotency factors SOX2 and NANOG as well as the PrE-specific transcription factors (TFs) GATA6 and SOX17, combined with high-resolution staging of embryos, allowed us to identify several distinct phases of lineage specification. We found that, similar to mouse, the process of lineage formation is temporally tightly controlled in rabbit, but rabbit embryos progress through some additional stages that are not apparent in the mouse. We noticed that restriction of GATA6 expression to a subset of rabbit ICM cells (PrE progenitors) did not coincide with establishment of mutually exclusive NANOG expression, suggesting that lineage formation in the rabbit ICM does not rely on mutual inhibition between these two factors. In contrast to mouse, manipulating FGF4 signalling in rabbit embryos did not affect the distribution of early PrE precursors, GATA6-positive cells, or lead to expansion of EPI compartment. In addition, modulation of the FGF pathway severely affected the expression of the late PrE marker SOX17 and the core pluripotency factor SOX2.

## RESULTS

### Accurate staging system for preimplantation rabbit embryos

Previous analyses of EPI and PrE specification in species other than the mouse have been performed mainly on embryos developing *in vitro* (reviewed by Piliszek et al., 2016). To collect data unaffected by *in vitro* culture conditions, we analysed formation of the two ICM lineages in freshly recovered rabbit embryos from natural matings.

**Fig. 1.**
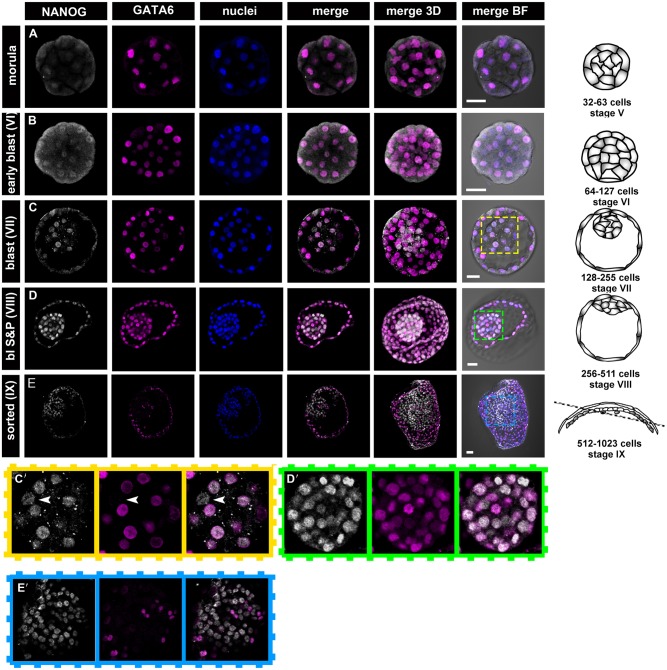
**Localisation of NANOG and GATA6 at the consecutive stages of development in rabbit embryos.** (A,B) NANOG and GATA6 are detected in all cells of the late morula and stage VI blastocyst. (C-D′) At stages VII and VIII, NANOG is still present in all ICM cells, whereas GATA6 is absent from some ICM cells (arrowheads in C′ indicate GATA6-negative cells; both factors are still present in the TE). C′ and D′ show magnifications of the boxed areas (ICM) in C and D, respectively. (E,E′) In stage IX blastocysts, GATA6 and NANOG become mutually exclusive in the majority of ICM cells, and the PrE and EPI cells become sorted into separate compartments. E′ shows magnification of the boxed area (ICM) in E. Each row represents a single optical section of one embryo and is accompanied by a 3D composite reconstruction of a *z*-stack (3D merge) and a schematic representation of an embryo at the corresponding stage (drawings not to scale). Dotted line indicates the section plane. Confocal images in A-D and all schematic drawings represent side view of the embryo, confocal image in E represents top view of the embryo (note that the embryo is folded owing to its large size, which partially obscures the TE). BF, brightfield; white, NANOG; magenta, GATA6; blue, Hoechst (nuclear marker). Scale bars: 50 μm.

**Fig. 2.**
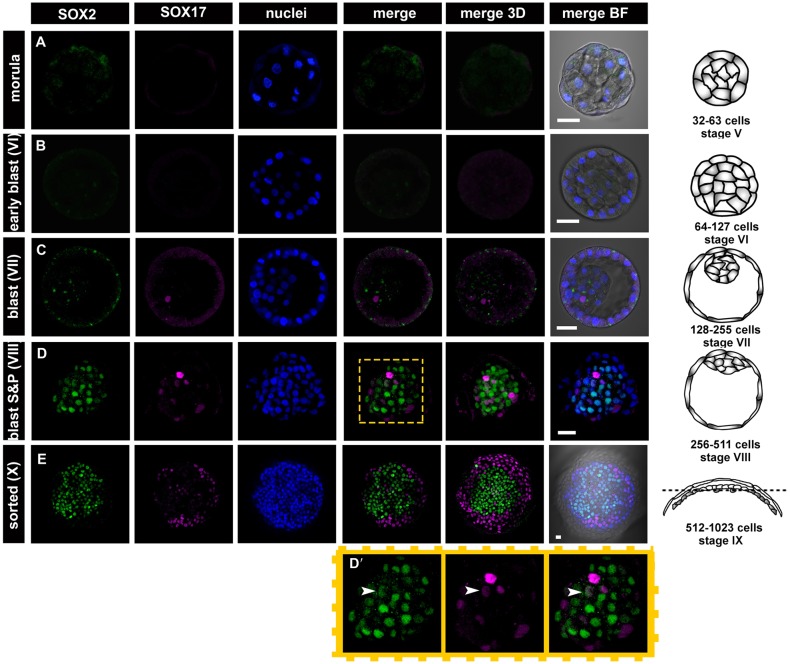
**Localisation of SOX2 and SOX17 at consecutive stages of development in rabbit embryos.** (A,B) SOX2 and SOX17 are absent in the morula and early blastocyst stage. (C,D) SOX17 is first detected in single ICM cells of stage VII blastocysts (C), and SOX2 in the ICM of the stage VIII blastocysts (D). D′ shows magnification of the boxed area (ICM) in D. Arrowheads indicate double-positive cell. (E) In the stage X blastocyst, the majority of SOX17-positive cells have become sorted into a ring surrounding SOX2-positive cells. Each row represents a single optical section of one embryo and is accompanied by a 3D composite reconstruction of a *z*-stack (3D merge) and a schematic of an embryo of the corresponding stage (drawings not to scale). Dotted line indicates the section plane. Confocal images in A-C and all schematics represent side view of the embryo, confocal images in D,E represent top view of the embryo. BF, brightfield; green, SOX2; magenta, SOX17; blue, Hoechst (nuclear marker). Scale bars: 50 μm.

Rabbit blastocyst stages encompass embryos of around 60-5000 cells and include several stages that do not have a mouse equivalent (Figs 1, 2 and 6). To analyse embryos properly at a comparable developmental stage, we employed a system of staging based on a total cell number in the embryo, which corresponds to the number of cell division rounds that the embryo has undergone (Table S3, Figs 1 and 2 and Fig. S1). This system includes the following stages: stage I, embryos after the first cell division [two cells, ∼24 h post-coitum (hpc)]; stage II, embryos after the second cell division (four cells, ∼30 hpc); stage III, eight cells (∼36 hpc); stage IV, 16-31 cells [∼2 days post-coitum (dpc)]; stage V, 32-63 cells (morula, ∼2.5 dpc); stage VI, 64-127 cells (compact morula or early cavitating blastocyst, ∼3 dpc); stage VII, 128-255 cells (∼3.25 dpc); stage VIII, 256-511 cells (∼3.5 dpc); stage IX, 512-1023 cells (∼3.75 dpc); stage X, 1024-2047 cells (∼4 dpc); stage XI, 2048-4095 cells (∼5 dpc); stage XII, ≥4096 cells (∼6 dpc). Our observations established that EPI versus PrE differentiation and sorting in the rabbit embryos takes place in blastocysts consisting of ∼100-1000 cells (3-4 dpc, stage VI-IX). Here, we present a detailed analysis of embryonic stages in rabbit development from stage IV morula until stage IX blastocysts, when lineages were clearly physically separated (Figs 1 and 2, Fig. S1).

### Restriction of GATA6 to PrE progenitors does not coincide with restriction of NANOG to EPI progenitors in rabbit

Establishment of mutual inhibition between the pluripotency factor NANOG and the endoderm-specific transcriptional regulator GATA6 has been reported to be a key event leading to initial segregation of EPI and PrE in mouse (reviewed by Chazaud and Yamanaka, 2016). Therefore, we used whole-mount immunofluorescence to analyse the expression dynamics of NANOG and GATA6 at consecutive stages of EPI and PrE formation.

GATA6 was detected in all nuclei of rabbit morulae and early blastocysts (Fig. 1A,B; stages IV-VI, *n*=11) and overlapped with NANOG in all cells, resembling the distribution observed in mouse morula and early blastocysts (Plusa et al., 2008). Partially overlapping expression of GATA6 and NANOG was also observed in stage VII and VIII rabbit blastocysts (Fig. 1C-D′; *n*=10 and *n*=7, respectively). However, we noticed a few GATA6-negative/NANOG-positive cells in the ICM of stage VII blastocysts (Fig. 1C,C′). GATA6-negative cells constituted on average 13.5% of total ICM cells (*n*=19). By stage VIII, the proportion of GATA6-negative cells increased to an average of 33.9% of all ICM cells (*n*=21). In both stage VII and VIII blastocysts, all ICM cells remained NANOG positive (Fig. 1C,D). Thus, downregulation of GATA6 in some of the ICM cells (presumably EPI progenitors) in rabbit blastocysts is not accompanied by downregulation of NANOG in other cells (presumably PrE progenitors). This result is in contrast to previously published data in the mouse system, where downregulation of NANOG in PrE progenitors at the blastocyst stage occurs in synchrony with downregulation of GATA6 expression in EPI progenitors and where establishment of mutual inhibition between NANOG and GATA6 was proposed to be one of the key events driving EPI versus PrE specification (Singh et al., 2007; Bessonnard et al., 2014). Rapid downregulation of NANOG in GATA6-positive cells was observed at stage IX (Fig. 1E,E′; *n*=7), with NANOG expression being retained predominantly in a GATA6-negative subset of ICM cells. Only a small number of cells remained double-positive (GATA6+/NANOG+). Mutually exclusive GATA6 and NANOG expression in the ICM of stage IX blastocysts was observed either in the form of a mosaic (salt-and-pepper) pattern (*n*=1) or in partially and/or completely sorted EPI and PrE cell populations, with GATA6-positive cells forming a ring encircling NANOG-expressing cells (Fig. 1E; *n*=6). Even after partial lineage segregation and formation of a ring of PrE, we observed a low percentage of double-positive cells (6.7% of all ICM cells).

Stages following PrE and Epi segregation at stage IX were not examined in detail; however, after blastocyst stage X, we found GATA6-positive cells in both TE and PrE derivatives of rabbit embryos, but not in EPI cells (*n*=2). This is consistent with the detection of *GATA6* mRNA in rabbit embryos at 2-6 dpc (stage IV-XII; Fig. S2B). Moreover, we did not observe any NANOG-positive cells after stage XI (*n*=5; Fig. S2C), which is consistent with the lack of *NANOG* mRNA in rabbit embryos collected at later stages (5 and 6 dpc) (Fig. S2A). Similar downregulation of *Nanog* mRNA and protein in the EPI lineage has been observed in implanting mouse embryos, at the stages directly following PrE and EPI segregation (stages between 4.5 and 4.75 dpc; Plusa et al., 2008; Acampora et al., 2016; Chambers et al., 2003; Saiz et al., 2016).

In summary, our results suggest that mutual inhibition between NANOG and GATA6 is not necessarily involved in initiating EPI versus PrE specification in the rabbit.

### Early expression of SOX2 and the late PrE marker SOX17 is not interdependent in the rabbit

OCT4 drives alternate developmental programmes in the mouse embryo by switching SOX partners, leading to either an endodermal (in conjunction with SOX17) or pluripotent (in conjunction with SOX2) cell fate (Aksoy et al., 2013). Moreover, SOX2 and SOX17 were reported to be restricted to ICM cells that had initiated differentiation towards EPI and PrE, respectively (Artus et al., 2011; Wicklow et al., 2014). We therefore examined the corresponding stages of rabbit embryo development (stage IV-X) for the presence of SOX2 and SOX17 protein by immunofluorescence.

**Fig. 3.**
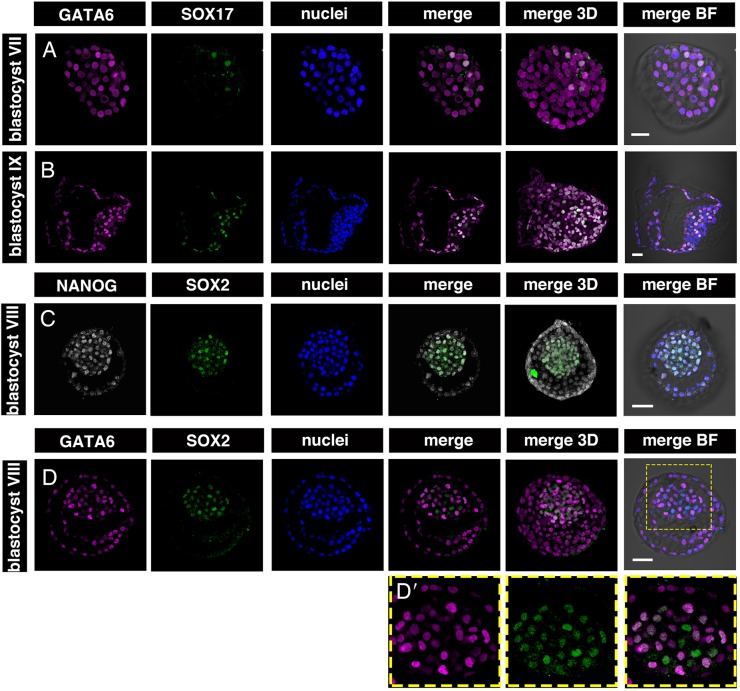
**Colocalisation of EPI and PrE markers in rabbit blastocysts.** (A) The PrE marker SOX17 is first detected in the ICM of the stage VII blastocyst, localising only to GATA6-positive cells. (B) As the blastocyst expands, the number of SOX17+/GATA6+ PrE cells increases, becoming spatially segregated from EPI in the stage IX blastocysts. Some of the SOX17+/GATA+ cells start migrating along the inner surface of the mural TE (weakly GATA6-positive TE cells are SOX17 negative). (C,D) The EPI marker SOX2 is first detected in the stage VIII blastocysts, colocalising with NANOG (C) and with the PrE-associated marker GATA6 (D) in some ICM cells. D′ shows magnification of the boxed area (ICM) in D. Each row represents a single optical section of one embryo. BF, brightfield; magenta, GATA6; white, NANOG; green, SOX17 (A,B) and SOX2 (C,D); blue, Hoechst (nuclear marker). Scale bars: 50 μm.

SOX2 and SOX17 nuclear localisation was mostly undetectable in blastocysts before stage VII (Fig. 2; 5/6 embryos). SOX17 first appeared at stage VII (*n*=15) in a small number of ICM cells (7%), whereas in all but one embryo at this stage, all cells were still SOX2 negative (Fig. 2C; *n*=11). By stage VIII, in almost all (13/14) embryos analysed, the ICM contained roughly equal proportions of SOX2-positive and SOX2-negative cells (Fig. 2D,D′). Only one embryo in this group (1/14) contained no SOX2-positive ICM cells. At the onset of its expression, SOX2 was co-expressed with the PrE markers SOX17 (Fig. 2D; *n*=4; 32% of SOX17-positive cells expressed SOX2, 12.4% of SOX2-positive cells expressed SOX17) and GATA6 (Fig. 3D,D′; *n*=6; on average 43.7% of GATA6-positive ICM cells expressed SOX2, 65% of SOX2-positive ICM cells expressed GATA6), further suggesting that EPI and PrE programmes are initiated independently in a subset of ICM cells in the rabbit. A mutually exclusive pattern of SOX2 and SOX17 expression was established by stage IX (*n*=2), shortly followed by a clear physical segregation of EPI and PrE progenitors (Fig. 2E; stage X; *n*=4). Our results confirm that in the rabbit, similarly to the mouse, SOX2 and SOX17 show partial overlap at the onset of their expression, but later become restricted to a specific lineage (EPI or PrE, respectively).

### Sequential activation of PrE and EPI TFS during lineage specification

Sequential activation of endoderm-specific TFs (GATA6→SOX17→GATA4→SOX7) is believed to occur during formation of PrE in mouse (reviewed by Schrode et al., 2013). To test whether similar sequential activation takes place in rabbit, we investigated the distribution of SOX17-positive and GATA6-positive cells in rabbit blastocyst stages VII and VIII. SOX17 was initially detected in a subset of GATA6-positive ICM cells (Fig. 3A; *n*=19), but by stage IX, coincident with lineage sorting, its expression overlapped with GATA6 in all PrE cells (Fig. 3B). This confirms that in rabbit, as in mouse, SOX17 expression is initiated in GATA6-positive PrE precursors in a sequential fashion. We also confirmed that at the onset of SOX2 expression, all SOX2-positive cells are also NANOG positive (Fig. 3C; stage VIII; *n*=1).

### Sustained inhibition of the ERK signalling blocks PrE formation but is not sufficient to expand the SOX2-positive compartment in rabbit embryos

**Fig. 4.**
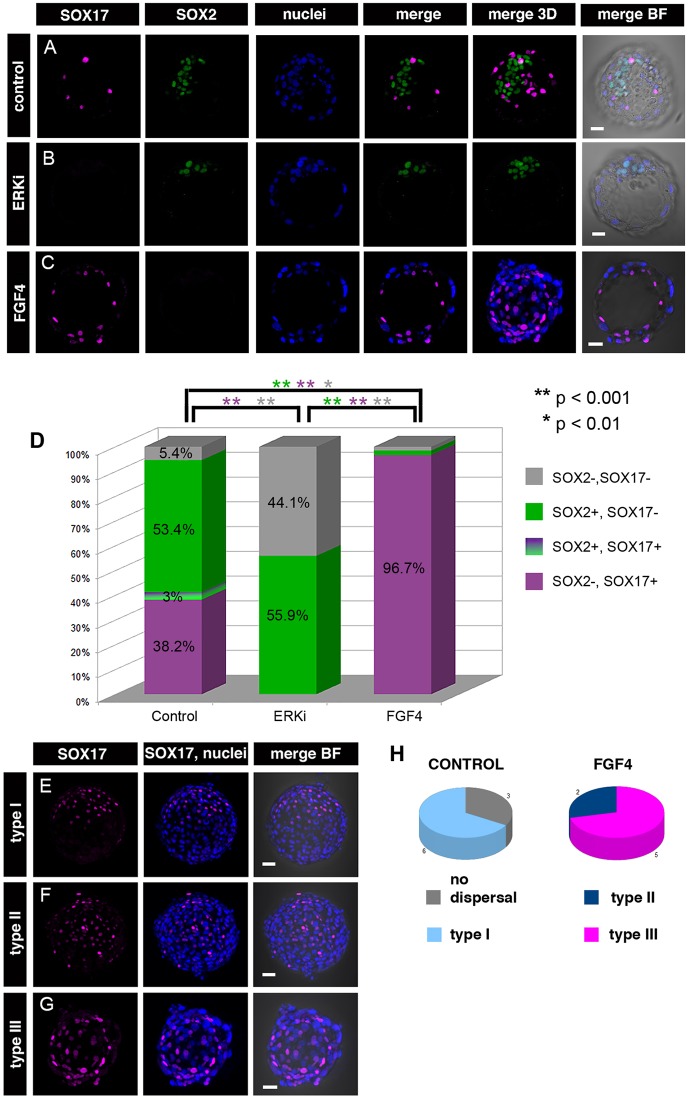
**Effects of FGF/ERK inhibition and activation in rabbit preimplantation development.** Immunolocalisation of PrE and EPI markers in rabbit blastocyst after *in vitro* culture. (A) Control embryos form ICM, correctly specifying and sorting SOX17-positive PrE cells and SOX2-positive EPI cells. (B) Embryos cultured in the presence of MEK/ERK inhibitor (ERKi) from compacted morula onward are devoid of SOX17-positive cells, with ICMs containing SOX2-positive and some double-negative (SOX2−, SOX17−) cells. (C) No SOX2-positive cells or inner cell mass are found in embryos cultured in the presence of FGF4, whereas SOX17-positive cells were spread underneath the TE. Each row of images represents a single optical section of one embryo. BF, brightfield; magenta, SOX17; green, SOX2; blue, Hoechst (nuclear marker). (D) Quantification of ICM contribution of SOX17+ and SOX2+ cells in control, ERKi-treated and FGF4-treated embryos. Percentage contribution of each cell type is indicated. Statistically significant differences between groups are marked by colour-coded asterisks, *P* values are as indicated (Z-test). (E-G) Distribution of SOX17-positive cells in FGF4-treated embryos. (E) Type I: SOX17-positive cells assembled on one pole of the embryo forming a continuous layer. (F) Type II: SOX17-positive cells dispersed underneath the TE and covering not more than half of the inner surface of the blastocoel. (G) Type III: SOX17-positive cells covering the whole cavity. (H) Pie charts representing dispersal of SOX17+ cells in control and FGF4-treated embryos. Scale bars: 50 μm.

**Fig. 5.**
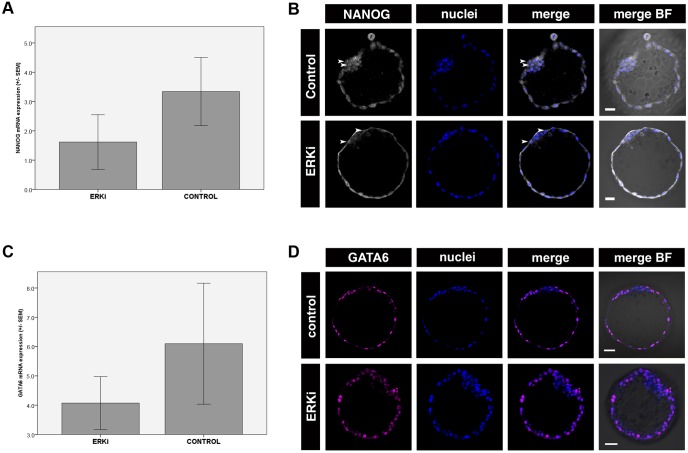
**Effects of FGF/ERK inhibition and activation on GATA6 and NANOG expression and localisation in rabbit preimplantation development.** (A,C) Expression levels of *NANOG* (A) and *GATA6* (C) mRNA in control and ERKi-treated rabbit embryos after *in vitro* culture. Error bars represent s.e.m. (B,D) NANOG (B) and GATA6 (D) distribution in control and ERKi-treated rabbit embryos. Each row represents a single optical section of one embryo. BF, brightfield; white, NANOG; blue, Hoechst (nuclear marker). Scale bars: 50 μm.

In mouse, specification of PrE from the bi-potent ICM depends on FGF/ERK signalling and, in the absence of this signal, the entire ICM acquires EPI identity. Use of small molecule inhibitors to block the FGF/ERK pathway in mouse resulted in preimplantation embryos depleted of PrE cells (Nichols et al., 2009). To verify whether the role of FGF/ERK signalling in the formation of the first lineages is conserved in mammals, we inhibited ERK phosphorylation (and subsequent activation of the ERK pathway) in cultures of stage V rabbit morula, using a selective MEK inhibitor (PD0325901, henceforth referred to as ERKi). Although SOX2 (SOX2+/SOX17−; EPI)- and SOX17 (SOX17+/SOX2−; PrE)-positive cells were readily identified in control embryos (Fig. 4A; *n*=9), no SOX17-positive cells were observed in ERKi-treated embryos (Fig. 4B,D; 0/241 ICM cells in 13 embryos). This result suggests that in the rabbit, similarly to the mouse, formation of a SOX17-positive PrE population requires FGF/ERK signalling. ERKi treatment did not increase the proportion of SOX2-positive cells in the ICM (55.9%, *n*=547 ICM cells in 13 embryos), in comparison with the control embryos (53.4%, *n*=670 ICM cells in 9 embryos; Fig. 4D). Similarly, inhibiting ERK phosphorylation did not alter the proportion of NANOG-positive ICM cells (2.5%, *n*=38 cells in 8 ERKi-treated embryos, in comparison with control embryos, 2.3%, *n*=41 cells in 8 embryos; Fig. 5B) or prevent downregulation of *NANOG* mRNA (Fig. 5A). A substantial reduction in the number of NANOG-positive cells in both control and experimental group was consistent with the reduction of NANOG-positive cells observed in freshly flushed embryos after stage X (Fig. S2C). This is in contrast to mouse studies, where inhibition of FGF/ERK signalling results in expansion of the EPI compartment as a result of all ICM cells converting to pluripotency, and prevents downregulation of NANOG observed in non-treated embryos after embryonic day (E) 4.5 (Nichols et al., 2009; Chambers et al., 2003; Saiz et al., 2016). Therefore, we conclude that in rabbit embryos, blocking PrE differentiation signals mediated by ERK kinase is not sufficient to induce an EPI identity. Instead, we observed an increase in the proportion of double-negative (SOX2−, SOX17−) cells in the ICMs of ERKi-treated embryos (Fig. 4C,D; non-treated control=5.4%; ERKi=44.1%). ERKi-treated rabbit embryos often contained a substantial number of ICM cells with nuclear fragmentation, which is associated with cell death (Fig. S3A), whereas in control embryos such cells were less frequent. Consistent with this, we observed a statistically significant reduction in ICM cell number in ERKi-treated embryos in comparison with control embryos (Fig. S3B; mean ERKi ICM cells=42.1, mean control ICM cells=74.4; Mann–Whitney, *P*<0.05). By contrast, TE cell number did not differ significantly between ERKi-treated and control groups (mean ERKi TE cells=241, mean control TE cells=293.2; Mann–Whitney, *P*>0.05), nor did we observe any fragmented nuclei in the TE of rabbit embryos treated with ERKi, suggesting that inhibition of FGF/ERK signalling has no detrimental effect on the survival of TE cells. In order to confirm whether ICM cells in ERKi-treated embryos were more prone to apoptosis, we tested ERKi and control embryos for the presence of active caspase 3 to detect apoptotic nuclei (Fig. S3C; Makarevich et al., 2008). Our data confirmed that the percentage of caspase-positive cells differs significantly between ICMs of ERKi-treated (10%; *n*=11) and control (7.3%; *n*=10) embryos (Z-test, *P*<0.01). No caspase activity was detected in TE in any of the embryos, which was consistent with the lack of visible nuclear fragmentation in TE cells observed in earlier experiments. Taken together, our results suggest that blocking FGF/ERK signalling in rabbit embryos prevents the formation of mature PrE, but does not transform all ICM cells into EPI.

### Sustained inhibition of ERK signalling does not affect distribution of the early PrE marker GATA6 in rabbit embryos

In mouse embryos, interfering with the FGF/ERK pathway affects the distribution of both early and late markers of PrE (Kang et al., 2013; Nichols et al., 2009). Conversely, in human embryos, distribution of early PrE marker GATA6 is not affected by ERK inhibition (Kuijk et al., 2012). We analysed GATA6 localisation in control and ERKi-treated embryos (*n*=12; Fig. 5D). Although ERK inhibition had a profound effect on later PrE markers, GATA6 distribution was unaffected after ERKi treatment, mirroring the data from human embryos. Consistent with this observation, *GATA6* mRNA levels were not significantly different between ERKi-treated and control embryos (Fig. 5C). In summary, inhibition of the FGF/ERK pathway affects PrE maturation, but does not affect distribution of the early PrE marker GATA6 in rabbit.

### FGF/ERK signalling controls the size of the SOX17-positive population in rabbit embryos

FGF4 is a potent activator of ERK signalling in the mouse and has been proposed to be involved in PrE specification and maintenance (reviewed by Hermitte and Chazaud, 2014). Our data on inhibition of the FGF/ERK pathway in rabbit embryos confirmed that the activity of ERK kinase is necessary for the formation and the survival of PrE precursors. To confirm whether FGF signalling is also involved in rabbit development, we first tested whether *FGF4* and two FGF receptors – *FGFR1* and *FGFR2* – are expressed in rabbit embryos before and during PrE-versus-EPI specification (Fig. S3D-F). We analysed samples of 2, 3, 4, 5 and 6 dpc embryos by qPCR. EPI-versus-PrE specification takes place at 3-4 dpc (stage VI-VIII; Fig. S1), and sorting at 4 dpc (stage IX-X). *FGF4* transcripts were present in rabbit embryos throughout the period of lineage specification. *FGFR2* transcripts decreased at 3 dpc, at the time of EPI and PrE specification (Fig. S3F), whereas *FGFR1* transcripts were present during the whole lineage specification period (2-4 dpc; Fig. S3E), suggesting that FGFR1 rather than FGFR2 might be responsible for transducing FGF signalling in rabbit embryos.

Next, we investigated whether stimulation of the FGF/ERK pathway is sufficient to induce PrE fate. The use of a saturating concentration of exogenous FGF4 is sufficient to divert all ICM cells towards PrE in mouse embryos (Yamanaka et al., 2010). To verify whether FGF4 addition to the culture medium has a similar effect on rabbit development, we cultured embryos from the morula stage in medium supplemented with FGF4, in parallel with non-treated control and ERKi-treated embryos. When we compared FGF4-treated embryos (*n*=7) with control embryos (*n*=9), we noticed a marked increase in the mean number of SOX17-positive (SOX17+/SOX2−) cells per embryo (Fig. 4D; 108.29 versus 28.44, respectively), whereas the mean number of SOX2-positive (SOX2+/SOX17−) cells was reduced (4/7 embryos) or absent (3/7; Fig. 4D; on average 2.0 cells in FGF4-treated embryos versus 39.8 in controls). Unlike in ERKi-treated embryos, we did not observe any increase in the number of double-negative cells or apoptotic nuclei. SOX17-positive cells accounted for the vast majority of ICM cells in FGF-treated embryos (Fig. 4D; 96.7% of total ICM in FGF4-treated embryos versus 38.2% in controls) and we did not observe any double-negative or double-positive cells. The mean total cell number and the mean ICM cell number did not exhibit statistically significant differences between control and FGF4-treated embryos (mean total FGF=377.6 versus mean total control=367.7, *P*=0.758; mean FGF ICM=112 versus mean ICM control=74.4, *P*=0.250). Therefore, we concluded that FGF4 treatment, unlike ERKi treatment, did not affect cell division or cell survival. As the survival of the ICM cells was not affected in FGF4-treated embryos, the expansion of the SOX17-positive compartment was most likely due to a preferential diversion of ICM cells to a PrE fate, rather than to a selective depletion of EPI progenitors or expansion of PrE progenitors.

In summary, our results confirm that, similarly to murine and bovine embryos, FGF4 treatment of rabbit embryos is sufficient to drive ICM cells towards a PrE fate.

### FGF4 treatment induces parietal endoderm identity and stimulates cell migration in rabbit embryos

Mouse embryos treated with FGF4 maintain normal blastocyst structure with a clearly distinguishable ICM on the embryonic side. In FGF-treated rabbit embryos, SOX17-positive cells were not assembled on one side of the embryo, as in the control blastocysts, but were spread underneath the polar TE (Fig. 4E-G), sometimes populating the whole inner surface of the blastocyst cavity (Fig. 4G, 5/9 embryos). We distinguished three distribution patterns for SOX17-positive cells (Fig. 4E-G): type I, defined as embryos with SOX17-positive cells assembled on one pole of the embryo forming a continuous layer (presumptive place where ICM was formed) (Fig. 4E); type II, defined as embryos with SOX17-positive cells dispersed underneath TE and covering not more than half of the inner surface of the blastocyst cavity (Fig. 4F); and type III, defined as embryos with SOX17-positive cells covering the whole cavity (Fig. 4G). Control embryos exhibited no dispersal (6/9) or little dispersal (3/9) whereas in the majority of FGF4-treated embryos, SOX17-positive cells were highly dispersed and were classified as type III (5/7) or type II (2/7) (Fig. 4H).

In contrast to mouse embryos (Bessonnard et al., 2014), SOX17-positive cells did not form a coherent ICM with epithelium forming at the cavity interface in FGF4-treated embryos. Instead, they acquired a spindle-shaped mesenchymal-like phenotype (Fig. 4C,G), mirroring the phenotype of the migratory parietal endoderm (PE) cells that can be found after differentiation of PrE to PE and visceral endoderm (VE) in mouse embryos. Therefore, we conclude that FGF4 treatment induced a PE-like phenotype in rabbit embryos and that the observed dispersion of SOX17-positive cells is consistent with a migratory phenotype of PE cells.

## DISCUSSION

Segregation of pluripotent EPI from extra-embryonic TE and PrE in mouse embryos has been studied extensively, yet the unifying principles of pre-implantation mammalian development remain largely unknown. Very few functional studies on PrE versus EPI specification in species other than the mouse have been performed to date (Boroviak et al., 2015; Kuijk et al., 2012; Roode et al., 2012; Nakamura et al., 2016). Because recent work highlights important disparities in pluripotent lineage specification across different mammalian species (reviewed by Kuijk et al., 2015; Piliszek et al., 2016), it is clear that establishing a unifying model of the origin of pluripotency and extra-embryonic lineage specification requires detailed investigations of how multiple mammalian species develop.

Here, we describe the formation of EPI and PrE lineages in the rabbit. We discovered significant differences in the mode of lineage specification between mouse and rabbit. Our results show that rabbit embryos mirror human embryonic development more closely than do mice.

In the mouse, restriction of *Nanog* and *Gata6* expression is accomplished in a seemingly co-dependent manner: upregulation of one factor is linked to downregulation of the other in a way suggestive of mutual inhibition (Bessonnard et al., 2014; Frankenberg et al., 2011; Schroeter et al., 2015; Singh et al., 2007; Xenopoulos et al., 2015). To understand the specification of PrE and EPI in the rabbit embryo, we evaluated the presence of NANOG and GATA6. Both TFs are initially present at the morula stage in all cells and eventually become restricted within the ICM to EPI (NANOG) and PrE (GATA6) lineages in rabbit embryos, similar to the mouse. However, unlike mouse, both factors persist in the TE long after the blastocyst cavity has formed and the ICM-derived lineages have become visibly segregated (until stage VIII for NANOG and beyond stage IX for GATA6). Persistence of GATA6 in the TE (even after spatial segregation of EPI and PrE) is also observed in human and non-human primate embryos (Boroviak et al., 2015; Roode et al., 2012). In rabbit embryos, downregulation of GATA6 in a subset of ICM cells appears to occur independently of NANOG, which is still present in the nuclei of both GATA6-negative and GATA6-positive cells in stage VII and VIII blastocysts. This indicates that in rabbit, differently from mouse, the initiations of EPI and PrE specification are not necessarily directly linked to each other and that levels of GATA6 and NANOG are not interdependent. It is possible that the mutual dependence of GATA6 and NANOG in the mouse is a rodent-specific mechanism. An alternative possibility, as a direct interaction between GATA6 and NANOG has yet to be demonstrated in mouse embryos, is that the GATA6-NANOG reciprocal relationship is not a part of the mechanism that specifies PrE versus EPI fate in mammals.

Recently, two members of the SRY-related HMG box family, SOX17 and SOX2, have been found in mouse to be more specifically associated with PrE and EPI than GATA6 and NANOG, respectively (SOX2: Avilion et al., 2003; White et al., 2016; Wicklow et al., 2014; SOX17: Artus et al., 2011; Blakeley et al., 2015; Morris et al., 2010). Our analysis of SOX2 and SOX17 localisation in preimplantation rabbit embryos confirmed their more restricted distribution compared with NANOG and GATA6. SOX2 expression in the rabbit is preceded by NANOG expression and is always restricted to the nuclei of cells already expressing NANOG. Moreover, whereas NANOG-positive cells have been found in both the ICM and TE of rabbit blastocysts, SOX2-positive cells were always restricted to the ICM. We therefore hypothesise that in the rabbit, initiation of SOX2 expression in a subpopulation of NANOG-positive cells might be an early sign of initiation of the EPI developmental programme. It is, however, important to note that at the onset of SOX2 expression, the presence of this marker is not necessarily associated with full EPI commitment, as nearly 25% of SOX17-positive cells and 40% of GATA6-positive ICM cells were also SOX2 positive. Similarly, activation of the PrE developmental programme in rabbit embryos does not appear to require downregulation of NANOG or a complete repression of the EPI developmental programme, as underscored by the presence of SOX2/SOX17 double-positive cells as well as GATA6/SOX2-positive cells. This is in contrast with mouse data, suggesting that the initiation of EPI and PrE maturation programmes occurs in an interdependent manner, and cells that upregulate EPI-specific genes, such as *Nanog* and *Sox2*, would downregulate or fail to initiate expression of PrE-specific markers, such as GATA6 and SOX17 (Chazaud et al., 2006; Guo et al., 2010; Plusa et al., 2008). In rabbit embryos, SOX17 can be detected at the stage when NANOG is still present in all ICM cells, but no clear negative correlation between SOX2 and SOX17 distribution is observed during the early stages of lineage specification.

**Fig. 6.**
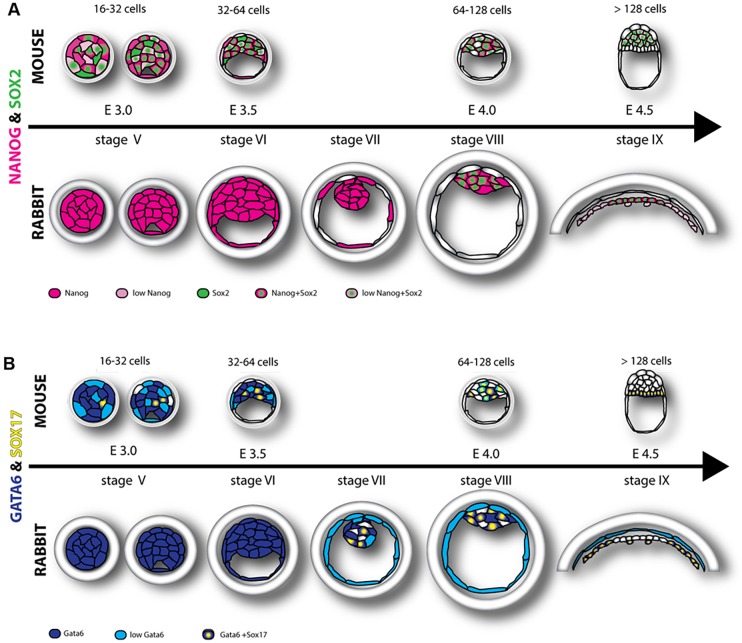
**Multi-step model of EPI/PrE lineage formation in mouse and rabbit embryos.** (A) In mouse embryos, the EPI markers NANOG and SOX2 are initially expressed in all blastomeres (16-32 cells), becoming restricted to EPI precursors distributed in a mosaic pattern within the ICM at around the 64-cell stage and sorting into EPI compartment at around the 120-cell stage, when NANOG is downregulated. In rabbit embryos, compact morulae (stage V) and blastocysts up to stage VII express NANOG, but not SOX2, in all cells. At stage VIII, SOX2 expression initiates in the majority of the ICM cells, and nearly all ICM cells still express NANOG. At stage IX, all EPI precursors express SOX2 and NANOG, concomitant with EPI and PrE cell sorting. (B) In mouse embryos, the PrE marker GATA6 is initially expressed in all of the cells. SOX17 becomes expressed in a few GATA6-positive cells in the ICM. At around the 64-cell stage, GATA6+/SOX17+ cells are distributed in a mosaic fashion in the ICM, later on sorting into the PrE compartment adjacent to the blastocoel cavity. In rabbit embryos, GATA6 is also initially expressed in all of the cells in morula, up to stage VI blastocyst. At stage VII, SOX17 expression initiates in some of the GATA6-positive ICM cells. At stage VIII, the proportion of GATA6/SOX17 double-positive cells as well as the proportion of GATA6-negative cells in the ICM increases. At stage IX, GATA6 and SOX17 are fully colocalised in the PrE and absent from EPI, while the ICM flattens and two compartments become sorted, with PrE encircling the EPI.

Previously, we identified three distinct phases of cell behaviour and gene expression in mouse embryos, from the morula stage until overt PrE formation at the peri-implantation blastocyst stage (Plusa et al., 2008; Fig. 6). The data presented in this manuscript suggest that lineage specification in rabbit embryos follows the same basic sequence of progression as in the mouse, although the timing of developmental events (defined by time since fertilisation as well as by cell number) differs between these species. We identified three consecutive stages of ICM lineage development common to both species: an overlapping expression phase (when factors characteristic to both EPI and PrE are expressed in all cells of the embryo); a refining phase (establishment of a salt-and-pepper pattern of EPI and PrE progenitor distribution); and a sorting phase (EPI and PrE progenitors segregated into two spatially separated layers). The length of the phases differed between species, but their consecutive order was the same. In the mouse, the establishment of a mutually exclusive salt-and-pepper pattern of EPI- and PrE-specific gene expression occurs during a single cell cycle after blastocyst formation (which, as discussed earlier, might reflect a co-dependence of initiation of EPI and PrE programmes in the mouse, or a timescale that does not allow resolution of two independent events), whereas in the rabbit, this process spans several cell cycles, with the PrE marker GATA6 being downregulated first, followed by NANOG downregulation a few cell cycles later (Fig. 6). Despite differences in the length of the refining phase, subsequent cell sorting in both species is achieved within a single cell cycle when the fully mutually exclusive pattern of EPI and PrE expression is established. This poses the question of whether these two processes are interlinked.

Currently, it is not clear whether the three phases of ICM lineage maturation are common to all mammalian species, though some evidence suggests that they might occur in human and other primates (Boroviak et al., 2015; Nakamura et al., 2016; Petropoulos et al., 2016).

Multiple studies in mouse embryos demonstrated that PrE specification depends on FGF signalling. It was postulated that, in the mouse, FGF4 expressed solely by EPI cells acts upon FGFR2 (Bessonnard et al., 2014; Guo et al., 2010; Kang et al., 2013). Our results show that *FGFR2* is expressed at very low levels during PrE and EPI specification in rabbit embryos, whereas *FGFR1* is present during the whole period of lineage specification. We therefore hypothesise that in rabbit development it is an FGF4-FGFR1 interaction that drives PrE specification. Interestingly, two recent reports demonstrated that although FGFR2 is specifically expressed in PrE, it is the pan-ICM-expressed FGFR1 that is crucial for establishment of PrE identity (Kang et al., 2017; Molotkov et al., 2017). Similarly, during pig development, *FGFR2* is not expressed at blastocyst stage whereas *FGFR1* is clearly detectable (Fujii et al., 2013) suggesting that dependence on FGFR1 to activate PrE programme might be more common in mammals.

Blocking the FGF/ERK pathway in mouse embryos forces all ICM cells to adopt an EPI identity (reviewed by Chazaud and Yamanaka, 2016). Unlike in mouse, chemical interference of FGF signalling during development of bovine and pig embryos fails to block PrE (hypoblast) formation and inhibition of the downstream pathway component MAPK/ERK kinase (MEK) only partially blocks PrE formation (Kuijk et al., 2012; Rodriguez et al., 2012). In human embryos, PrE formation is not blocked by FGF receptor inhibition or ERK inhibition (Roode et al., 2012). In rabbit, inhibition of ERK does not influence distribution of the early PrE marker GATA6, similar to human embryos (Kuijk et al., 2012), but it has a pronounced effect on the expression of the late PrE marker SOX17. In the mouse, initiation of GATA6 expression in *Fgf4* mutant embryos is unaffected but FGF4 is necessary for maintenance of GATA6 after the initiation of lineage segregation (Kang et al., 2013; Ohnishi et al., 2014). It is possible that in the mouse the transition from FGF-independent to FGF-dependent GATA6 expression is faster and more abrupt, whereas in other mammalian species this time frame is extended. This could potentially account for the lack of effect on GATA6 expression in human and rabbit embryos treated with FGF pathway inhibitors (Kuijk et al., 2012; present study). It is possible that all mammals depend on FGF signalling for at least some aspects of PrE specification and/or maturation. However, the timing of the initiation of PrE formation and the window of FGF responsiveness may differ substantially between different species.

We did not observe any marked increase in the number of SOX2-positive EPI cells in rabbit embryos treated with MAPK/ERK kinase inhibitor in comparison with control embryos. Instead, the ICMs of ERKi-treated embryos contained substantial number of SOX2 and SOX17 double-negative cells. This result suggests that blocking FGF signalling is not sufficient to induce an EPI fate in the rabbit and that some additional signal may be required. Therefore, we speculate that in rabbit embryos EPI formation is driven by two independent events: the upregulation of pluripotency genes in a subset of ICM cells and the concomitant loss of responsiveness to FGF signalling. The failure to initiate any component of this programme would result in either double-negative cells (failing to upregulate pluripotency genes) or double-positive cells (upregulating the pluripotency network but also responsive to FGF signalling). In our model, double-negative cells that fail to make an appropriate cell fate choice are eliminated from the ICM population by apoptosis. Supporting this notion, double-negative cells (SOX2−, SOX17−) were found with much higher frequency in ERKi-treated embryos and cell death was much more pronounced in those embryos compared with the control.

Although specification of EPI seems to differ between mouse and rabbit, providing an excess of exogenous FGF4 in both species results in development of embryos with all ICM cells expressing solely PrE markers (Yamanaka et al., 2010; present study). Notably, treatment of rabbit embryos with FGF4 induced a migratory phenotype leading to dispersion of SOX17-positive cells underneath the TE, in some cases covering the whole inner surface of the blastocyst cavity. Moreover, these highly migratory SOX17-positive cells acquired a spindle-shaped mesenchymal-like phenotype, resembling mouse migratory PE cells. In the mouse, soon after implantation PrE differentiates into VE, which forms an epithelium enveloping the growing post-implantation EPI, and PE, a mesenchymal population of cells disseminating from the original PrE epithelium and migrating along the inner surface of the TE (Enders et al., 1978; Gardner and Papaioannou, 1975). Because FGF4 has previously been suggested to play a role in regulation of cell migration (Webb et al., 1997; Jeong et al., 2016), we hypothesise that FGF4 treatment in rabbit embryos either directly induces a migratory PE fate in ICM cells or promotes an epithelial-to-mesenchymal (EMT)-like PrE-to-PE transition.

In summary, our data demonstrate the existence of several differences in pre-implantation development between rabbit and mouse. Importantly, lineage specification in rabbit resembles human, non-human primate and domestic animal development rather than mouse, posing the question whether murine development is the most representative example of mammalian development.

## MATERIALS AND METHODS

### Animals

Rabbits (*Oryctolagus cuniculus*, Popielno breed) were maintained under a 14-h light/10-h dark cycle in the facilities of The Institute of Genetics and Animal Breeding of the Polish Academy of Sciences (IGAB PAS) according to the institutional guidelines. Experimental procedures were approved by the Third Local Ethics Committee (Warsaw, Poland).

### Embryo collection and culture

Embryos were collected from natural matings by flushing the oviduct (1-3 dpc) or uterus (4-6 dpc) of donor females under general anaesthesia with pre-warmed medium (TCM-199+10% fetal bovine serum, Sigma). Where indicated, embryos were cultured *in vitro* from the zygote stage (18-20 h after mating) in drops of RDH medium (RPMI:DMEM:Ham's F10, Life Technologies, at 1:1:1) supplemented with 5 mM taurine and 0.3% bovine serum albumin (Jin et al., 2000), under mineral oil, in a humidified incubator, at 38.5°C, 5% CO_2_ in air. After 48 h of *in vitro* culture (morula stage), embryo coats were pre-digested by a 30-s incubation in 0.5% pronase. Where indicated, media were supplemented with 1 μM PD0325901 (MEK inhibitor, Stemgent) or with 100 ng/ml recombinant human FGF4 (R&D Systems) plus 1 μg/ml heparin (Sigma), and control embryo culture was supplemented with an equivalent amount of DMSO used as a solvent for the inhibitor stock solutions (0.01%; Sigma).

### Immunostaining

Embryos were fixed in 4% paraformaldehyde in PBS with 0.1% Tween-20 (Sigma) and 0.01% Triton X-100 (Sigma) for 20 min at room temperature. Embryo coats were removed mechanically after fixation (Puschel and Viebahn, 2010). Fixed embryos were immunostained as previously described (Plusa et al., 2008). Antibody details are listed in Table S2.

### Image analysis

Embryos were placed on a glass-bottom dish (MatTek) and visualised using a Nikon R1 confocal microscope. Analysis of images was performed using IMARIS software (Bitplane AG). For cell number count, nuclei were identified using the ‘spot’ option with an estimated diameter of 7-10 μm. The number of nuclei identified by IMARIS was confirmed manually. 3D confocal images were created by maximum intensity projection using the IMARIS software (Bitplane AG) ‘volume’ option.

### Embryo collection for gene expression analysis

*In-vivo*-obtained rabbit embryos were collected at successive developmental stages at 2, 3, 4, 5 and 6 dpc. The selected material was placed in a minimal volume of PBS in 1.5 ml tubes (low binding, Eppendorf), snap frozen in liquid nitrogen and stored at −80°C.

### RNA extraction and cDNA synthesis

Total RNA was extracted with the High Pure miRNA Isolation Kit (Roche Diagnostics) following the manufacturer's protocol, as previously described (Madeja et al., 2013). RNA quality and concentration was measured using a NanoDrop c2000 (Thermo Scientific) and for each sample, the reverse transcription reaction was performed on 100 ng of total RNA. cDNA synthesis was performed with the Transcriptor High Fidelity cDNA Synthesis Kit (Roche Diagnostics) following the manufacturer's protocol. The samples were stored at −20°C.

### Quantitative real-time PCR reaction

Quantitative PCR (qPCR) was performed on a Roche Light Cycler 96 instrument. Calculations of expression level were based on the standard curve method with three reference genes: *H2AFZ*, *HPRT1* and *YWHAZ* (Mamo et al., 2007). Each sample was analysed in triplicate with all of the primer sets chosen for the experiment. For each developmental stage we collected six independent samples. The primer pairs were designed to span introns (Table S1). The reactions were carried out as previously described (Madeja et al., 2013). Product specificity was confirmed by melting-point analysis and agarose gel electrophoresis.

### Statistical analysis

Analysis was performed using IBM SPSS Statistics 22.0 software. Before computing, all data were subjected to Kolmogorov–Smirnov tests for normal distribution. Statistical differences in gene expression level between developmental stages and differences in cell number between groups were calculated using Kruskal–Wallis test and nonparametric Mann–Whitney test. Differences in percentage of cells between groups was analysed using Z-tests. *P*<0.05 was considered statistically significant.

## Supplementary Material

Supplementary information
