# Supplementary material for: Suppression of ERK signalling abolishes primitive endoderm formation but does not promote pluripotency in rabbit embryo
Source: Development. 2017 Oct 15;144(20):3719–30. doi: 10.1242/dev.156406 (PMC5675450; doi:10.1242/dev.156406)
Supplement: Supplementary information [file develop-144-156406-s1.pdf]

Supplementary tables

Table S1. Primer pairs and real-time PCR reaction conditions

GENE	Sense primer	Antisense primer	Annealing temp	Product size(bp)
HPRT1	CAGGACTGAAAGGCTTGCTC	AATCCAGCAGGTCAGCAAAG	62	110
H2AFZ	GCCATCCTGGAGTACCTCAC	AGCAAGTTGCAAATGACGAG	62	102
YWHAZ	GGTCTGGCCCTTAACTTCTCTGTGTTCTA	GCGTGCTGTCTTTGTATGATTCT	62	142
NANOG	AGACAGAAATACCTCAGCCTTCA	AATTGTTTCTCTGCCACCTCTTA	60	127
GATA6	TGCGGCATCTACAGCAAGAT	TTTCTGCGCCATAAGGTGGT	66	134
FGF4	GGCGTGGTGAGCATCTTT	TACCTGTGGGACTCGTAGGC	67	152
FGFR1	TTGCCTGAACAAGATGCGCT	CTACGGGCTTACGGTTTGGT	58	109
FGFR2	ACGTTCAAGCAGTTGGTGG	CCTGAAAGAAGGGAAGAGAGACG	60	151

Table S2. List of antibodies used in this study

Primary antibody	host	company	Catalog number	dilution
anti-SOX2	rabbit	Abcam	ab97959	1:100
anti-SOX2	mouse	R&D systems	SC009 (kit)	1:100
anti-GATA6	goat	R&D Systems	AF1700	1:100
anti-SOX17	goat	R&D Systems	AF1924	1:100
anti-SOX17	goat	R&D Systems	NL557	1:100
anti-NANOG	rabbit	Peprotech	#500-P236	1:100
anti-NANOG	rabbit	Cosmo Bio	RCAB0001P	1:500
Secondary antibodies (Alexa Fluor) were purchased from Invitrogen or Abcam and used at 1:500 dilution.				

Table S3. Staging of preimplantation rabbit embryos

Stage	Cell number	Time post fertilisation	Morphology
I	2	16-22 hpc	2-cell stage
II	4	21-30 hpc	4-cell stage
III	8	1-1.5 dpc	8-cell stage
IV	16-31	1.5-2.5 dpc	morula
V	32-63	2.5-3.25 dpc	morula
VI	64-127	3.0-3.5 dpc	compact morula/ cavitating blastocyst
VII	128-255	3.0-3.75 dpc	blastocyst
VIII	256-511	3.25-3.75 dpc	blastocyst
IX	512-1023	3.5-4.25 dpc	blastocyst
X	>1024	3.75-4.25 dpc	blastocyst

SUPPLEMENTARY FIGURES

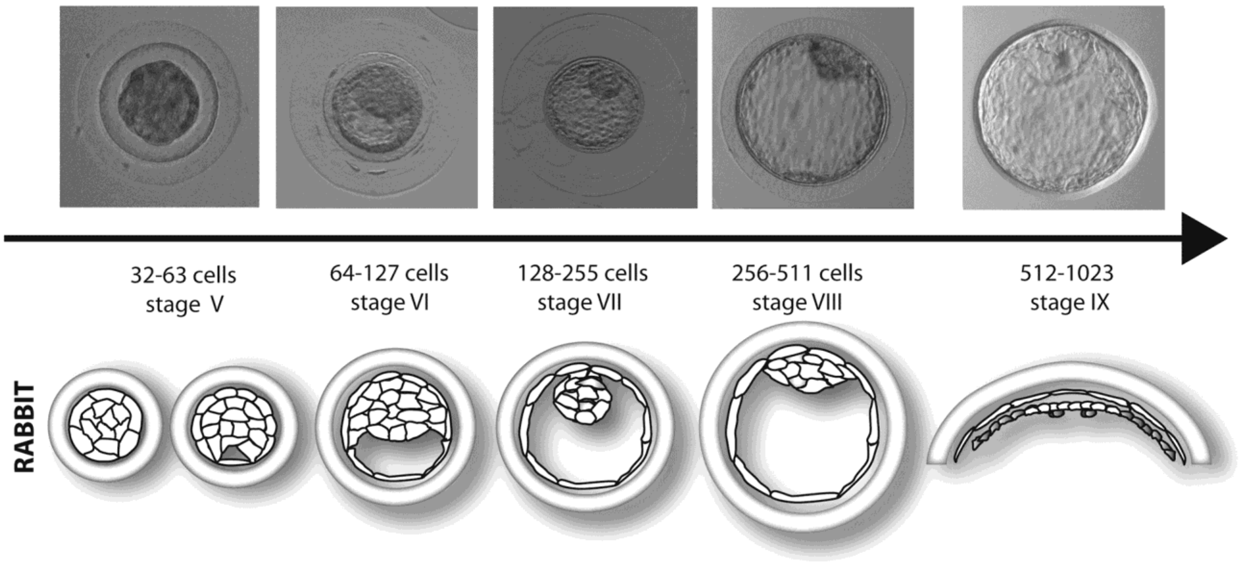

Fig. S1

Development of rabbit embryos during epiblast and primitive endoderm specification, and proposed staging system.

Upper row – single optical section, bright field images of rabbit embryos. Lower row – schematic representation of corresponding stages of rabbit embryo development. Embryos not to scale.

Development • Supplementary information

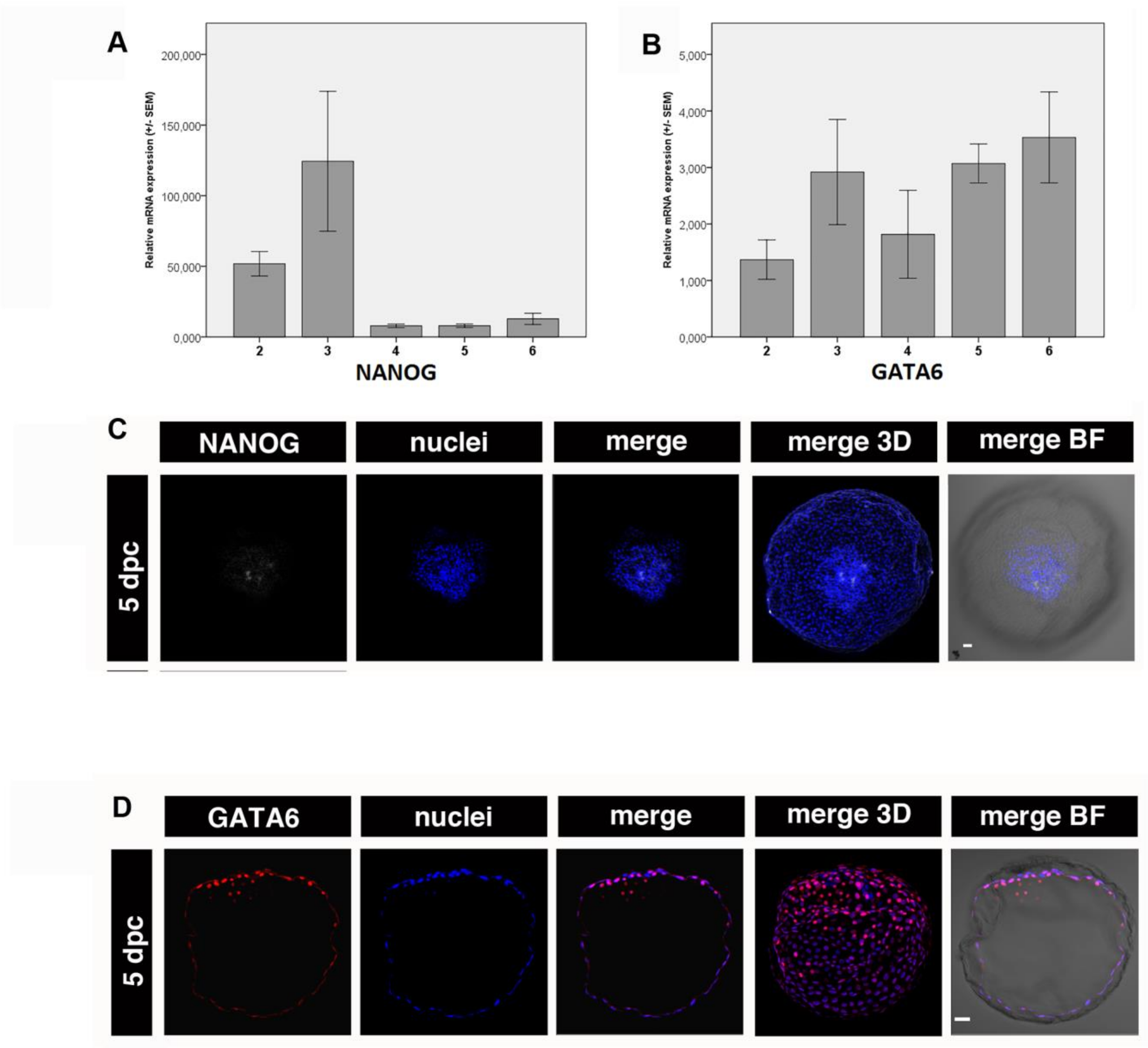

FIG. S2

(A-B) Expression levels of (A) *NANOG* and (B) *GATA6* in rabbit embryos at consecutive stages of development (2 - 6 dpc). Error bars represent SEM.

(C) NANOG and (D) GATA6 distribution in rabbit embryos at 5 dpc. Arrowheads indicate NANOG-positive cells. Each row represents a single optical section of one embryo. BF, bright field; white, NANOG; blue, Hoechst (nuclear marker). Scale bar: 50 µm.

Development • Supplementary information

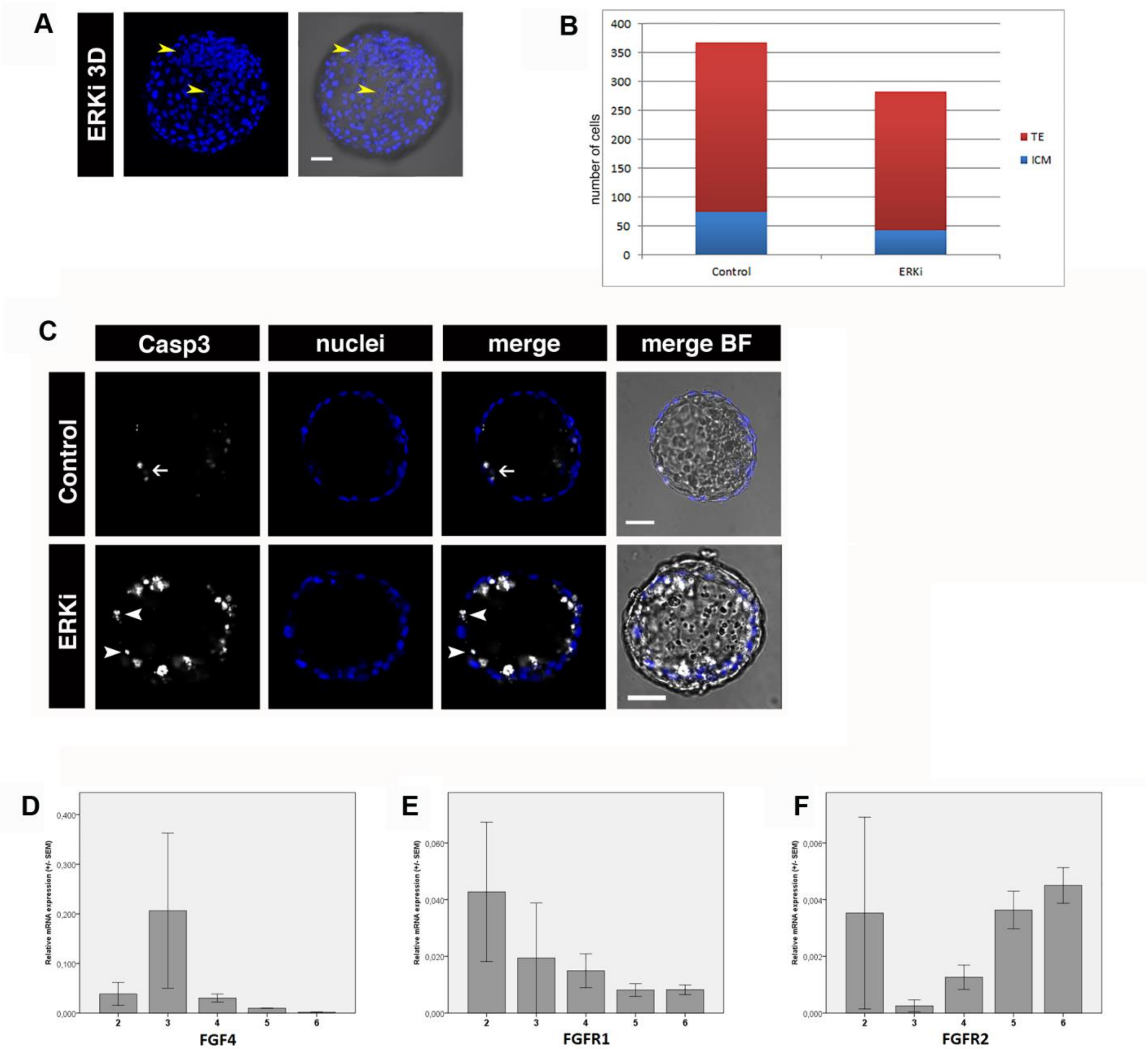

Fig. S3

(A) ERKi-treated embryos exhibit pronounced ICM cell death. Arrowhead marks cells undergoing nuclear fragmentation. BF, bright field; blue, Hoechst (nuclear marker). Scale bar: 50 μm.

(B) Differences in mean cell number and lineage contribution in control and ERKi treated rabbit embryos. TE (red) and ICM (blue).

Development • Supplementary information

(C) Cleaved Caspase 3 activity in control and ERKi-treated rabbit embryos. Each row represents a single optical section of one embryo. Arrowheads indicate caspase-positive cells. Arrows indicate apoptotic cell debris. BF, bright field; white, cleaved caspase 3; blue, Hoechst (nuclear marker). Scale bar: 50 µm.

(D-F) Expression levels of (D) *FGF4*, (E) *FGFR1* and (F) *FGFR2* in rabbit embryos at consecutive stages of development (E2.0 to E6.0) Error bars represent standard error of the mean (SEM).

Development • Supplementary information
